# Strain-Specific Effects of Early-Life Probiotic Supplementation on Respiratory Infections in Infants: A Systematic Review and Meta-Analysis

**DOI:** 10.3390/nu18132067

**Published:** 2026-06-24

**Authors:** Salvatore Michele Carnazzo, Emanuele Sinagra, Dario Raimondo, Arianna Sferruzza, Roberto Ajovalasit, Alessandro Vitello, Andrea Domenico Praticò, Marcello Maida

**Affiliations:** 1 Department of Medicine and Surgery, University of Enna “Kore”, 94100 Enna, Italy; alessandro.vitello@unikore.it (A.V.); andrea.pratico@unikore.it (A.D.P.); marcello.maida@unikore.it (M.M.); 2 Gastroenterology and Endoscopy Unit, Fondazione Istituto San Raffaele Giglio, 90015 Cefalù, Italy; emanuelesinagra83@googlemail.com (E.S.); dario.raimondo@hsrgiglio.it (D.R.); arianna.sferruzza@gmail.com (A.S.); roberto.ajovalasit01@gmail.com (R.A.)

**Keywords:** probiotics, synbiotics, respiratory tract infections, upper respiratory tract infection, infants, early-life microbiota, gut–lung axis, randomized controlled trials, systematic review, meta-analysis

## Abstract

Background/Objectives: Probiotic and synbiotic supplementation has been proposed as a preventive strategy against respiratory tract infections (RTIs) in early childhood, although evidence in infants and young children remains inconsistent. This systematic review and meta-analysis aimed to evaluate the effects of probiotic or synbiotic supplementation administered during the first 24 months of life on respiratory infection outcomes. Methods: PubMed/MEDLINE, Embase, and Scopus were systematically searched for randomized controlled trials published between January 2015 and 30 September 2025. Eligible studies included infants and children aged ≤24 months receiving oral probiotics or synbiotics compared with placebo, no intervention, or standard care. The primary outcome was the incidence of at least one upper respiratory tract infection (URTI), while the secondary outcome was the incidence of any RTI. Pooled odds ratios (ORs) and 95% confidence intervals (CIs) were calculated using random-effects models. Risk of bias was assessed using the Cochrane RoB 2 tool, and certainty of evidence was evaluated according to the GRADE approach. Results: Nine randomized controlled trials were included. Probiotic or synbiotic supplementation did not significantly reduce the risk of URTI (OR 0.95, 95% CI 0.47–1.95; I^2^ = 78%). A non-significant trend toward a reduced risk of any RTI was observed (OR 0.66, 95% CI 0.35–1.25; I^2^ = 69%). Exploratory subgroup analyses suggested possible strain-specific effects, with signals observed for *Bifidobacterium longum* subsp. *infantis* in relation to URTI prevention and *Lactiplantibacillus plantarum* ATCC 202195 for reduction in any RTI. However, these findings were based on a limited number of studies and should be interpreted cautiously. No serious adverse events attributable to supplementation were reported. Conclusions: Current evidence does not support the routine use of probiotic or synbiotic supplementation for the prevention of respiratory infections in children aged ≤24 months. However, potential strain-specific benefits warrant further investigation in adequately powered randomized trials.

## 1. Introduction

Respiratory tract infections (RTIs), including lower respiratory tract infections (LRTIs), remain a leading cause of morbidity and mortality in early childhood worldwide [1,2,3,4]. The gut microbiota plays a key role in immune maturation during early life, and disruptions in its development have been associated with increased susceptibility to infections [5,6,7]. Mechanistic evidence supports a gut–lung axis influencing immune responses to respiratory pathogens [8,9]. Probiotics have been proposed as a non-pharmacological strategy for RTI prevention in children [10]. A recent Cochrane review suggested reduced URTIs, although heterogeneity limits certainty [11]. In addition, several recent reviews and expert consensus documents have highlighted the importance of considering probiotic effects at the strain level and have emphasized the need for more targeted evidence in pediatric populations [12,13]. Current clinical guidelines therefore emphasize strain-specific effects and do not support routine use beyond gastrointestinal indications [14,15]. Most available evidence includes broad pediatric populations and rarely focuses on infants under two years of age. The first 24 months of life represent a critical developmental window for immune programming and microbiota maturation. During this period, microbial colonization patterns are particularly dynamic and strongly influenced by feeding practices, antibiotic exposure, and mode of delivery, factors that may modify susceptibility to respiratory infections and responses to probiotic interventions. In addition, many systematic reviews combine URTI with other respiratory outcomes, limiting clinical interpretability for primary prevention in early life [11,16]. Despite growing interest in probiotics as a preventive strategy against respiratory infections, previous meta-analyses have produced inconsistent conclusions because they pooled heterogeneous pediatric populations, probiotic strains, and respiratory outcomes. Consequently, clinicians still lack clear evidence regarding which probiotic strains may be effective, when supplementation should be initiated, and the magnitude of any potential benefit. Unlike previous meta-analyses that evaluated probiotics as a broad therapeutic category, the present study specifically aimed to investigate strain-dependent differences in efficacy within a narrowly defined early-life population. This strain-specific approach represents a necessary refinement of existing evidence and may provide more clinically actionable information than conventional pooled analyses. To address these knowledge gaps, we conducted a systematic review and meta-analysis restricted to children aged ≤24 months, evaluating URTI as the primary outcome and any respiratory infection as a secondary outcome, with additional strain-specific analyses. Safety remains relevant, particularly in high-risk infants where rare probiotic-associated sepsis has been reported [17,18,19].

## 2. Materials and Methods

### 2.1. Study Design and Registration

This systematic review and meta-analysis was conducted in accordance with PRISMA 2020 guidelines [20] and prospectively registered in PROSPERO (CRD4202021157621). The original protocol specified inclusion of infants ≤23 months; before final data synthesis, this threshold was aligned to ≤24 months to reflect standard pediatric classifications. This amendment was implemented before outcome extraction and quantitative synthesis. The rationale was to align the eligibility criteria with internationally accepted pediatric age classifications and to improve consistency with the age definitions used in the included trials. The modification did not affect study eligibility decisions or materially alter the pooled estimates. Additional prespecified refinements included separating upper respiratory tract infections (URTI) from any respiratory tract infection (RTI; URTI ± LRTI) and restricting quantitative synthesis to dichotomous outcomes to improve clinical interpretability.

### 2.2. Eligibility Criteria

Eligibility criteria were defined according to the PICO framework. The population included infants and young children aged ≤24 months without acute respiratory infection at enrollment. Trials with mixed pediatric populations were eligible if ≥80% of participants were ≤24 months. Interventions consisted of oral probiotics or synbiotics, regardless of strain, dose, duration, or formulation, administered for prevention of respiratory infections. No restrictions were applied regarding probiotic strain because current evidence suggests that probiotic effects are highly strain-dependent and cannot be generalized across species or genera. Therefore, all strains administered during the first 24 months of life were eligible, allowing subsequent strain-specific subgroup analyses to explore potential differences in efficacy. Comparators were placebo, no intervention, or standard care. The primary outcome was incidence of ≥1 URTI episode; secondary outcomes included any RTI (URTI or LRTI), number of infections, illness duration and severity, and absenteeism. Only randomized controlled trials, including cluster RCTs, were included. Non-randomized studies, therapeutic interventions, trials enrolling children >24 months, and non-English publications were excluded. Continuous outcomes were not pooled because of substantial heterogeneity.

### 2.3. Literature Search Strategy

PubMed/MEDLINE, Embase, and Scopus were searched from January 2015 to 30 September 2025. The search window was defined a priori to improve comparability across studies in terms of strain characterization, formulation, and outcome definitions. Search terms included MeSH and free-text keywords related to probiotics, synbiotics, respiratory infections, and randomized controlled trials. Reference lists of relevant reviews and included studies were also screened. Full search strategies are provided in the Supplementary Material.

### 2.4. Study Selection

**Figure 1:**
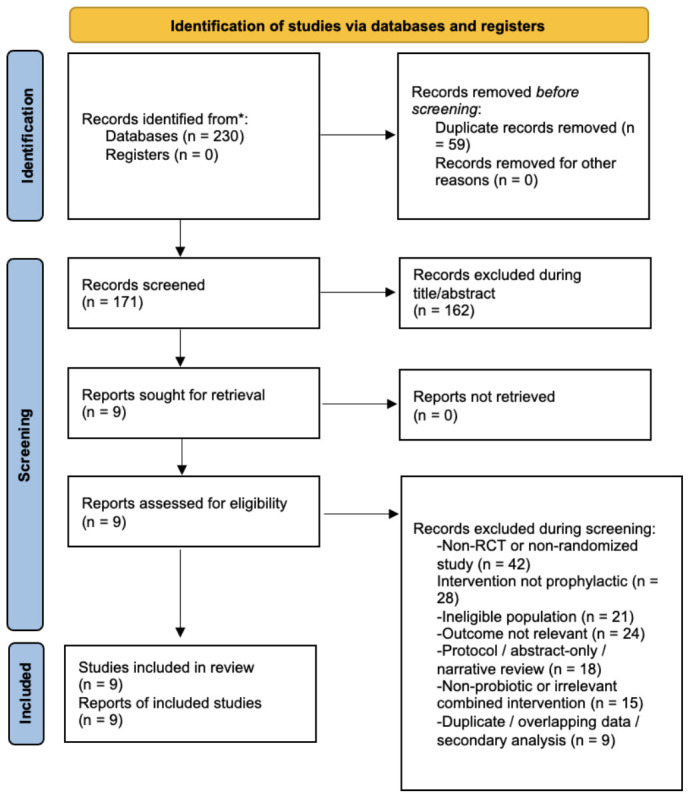
PRISMA 2020 flow diagram of study selection. Flowchart illustrating the identification, screening, eligibility assessment, and inclusion of randomized controlled trials evaluating probiotic or synbiotic supplementation for prevention of respiratory tract infections in children aged ≤24 months. Abbreviation: RCT, randomized controlled trial. *: Records identified from databases and registers according to the PRISMA 2020 flow diagram template.

Two reviewers independently screened titles and abstracts using Rayyan [21]. Potentially eligible full texts were assessed against predefined inclusion criteria. Disagreements were resolved by consensus or, when necessary, by a third reviewer. The study selection process is shown in Figure 1 (PRISMA 2020 flow diagram).

### 2.5. Data Extraction

Two reviewers independently extracted data using a pre-piloted standardized form. Extracted items included study characteristics, participant demographics, intervention and comparator details, outcomes, follow-up duration, and adverse events. When trials included multiple probiotic arms, data were extracted separately whenever feasible. All extracted data were cross-checked, and discrepancies were resolved by consensus.

### 2.6. Outcome Definitions

The primary outcome was incidence of at least one URTI during follow-up, defined as a physician-diagnosed upper respiratory tract infection. The secondary outcome, any RTI, included both upper and lower respiratory tract infections. Outcomes were analyzed separately to avoid clinical misclassification and to preserve interpretability across biologically distinct respiratory disease entities.

### 2.7. Risk of Bias Assessment

Methodological quality was assessed independently by two reviewers using the Cochrane Risk of Bias 2.0 tool [22]. Domains included randomization, deviations from intended interventions, missing outcome data, outcome measurement, and selection of reported results. Disagreements were resolved through discussion or consultation with a third reviewer. Overall risk-of-bias judgments were incorporated into subgroup and sensitivity analyses.

### 2.8. Data Synthesis and Statistical Analysis

Meta-analyses were conducted using Review Manager (RevMan) [23]. Cluster-randomized trials were incorporated using cluster-adjusted effect estimates reported by the original studies and analyzed with the generic inverse variance method. Random-effects models with the DerSimonian–Laird estimator were used to calculate pooled odds ratios (ORs) with 95% confidence intervals (CIs) [24]. ORs were selected to ensure consistency across studies, including those reporting only adjusted effect estimates. Statistical heterogeneity was assessed using Cochran’s Q and quantified with the I^2^ statistic [25]. Continuous outcomes were reported using heterogeneous definitions, measurement scales, and follow-up periods across studies. Preliminary assessment indicated that pooling through standardized mean differences would have introduced substantial clinical and methodological heterogeneity. Therefore, these outcomes were synthesized narratively [26]. The certainty of evidence for the primary and secondary outcomes was assessed using the GRADE approach [27], considering risk of bias, inconsistency, indirectness, imprecision, and publication bias. Separate GRADE assessments were performed for URTI and any RTI.

## 3. Results

### 3.1. Study Selection

A total of 230 records were identified from database searches. After removal of 59 duplicates, 171 titles and abstracts were screened. Of these, 162 were excluded due to ineligible design (n = 42), non-prophylactic interventions (n = 28), wrong population (n = 26), irrelevant outcomes (n = 24), abstracts or protocols only (n = 18), non-probiotic interventions (n = 15), or duplicate/overlapping data (n = 9). Ultimately, 9 randomized controlled trials (RCTs) met inclusion criteria and were included in the quantitative synthesis (Figure 1, PRISMA 2020 flow diagram).

### 3.2. Study Characteristics

Across all studies, participants were ≤24 months of age at baseline or in cohorts where ≥80% were under two years (Table 1). Interventions consisted of oral probiotic or synbiotic supplementation, administered as fortified milk, powders, or capsules/sachets. Treatment duration ranged from 4 weeks to 6 months, and follow-up periods from 2 to 12 months. Frequently evaluated strains included *Lactobacillus paracasei* F19, *L. rhamnosus* GG, *L. plantarum* ATCC 202195, *Limosilactobacillus fermentum* combined with GOS, *Bifidobacterium longum* subsp. *infantis*, and *B. animalis* subsp. *lactis*.

Most trials were double-blind, placebo-controlled parallel-group designs. The cluster-randomized synbiotic trial by Panigrahi et al. was incorporated using the cluster-adjusted effect estimate reported by the original authors.

### 3.3. Risk of Bias Assessment

**Figure 2:**
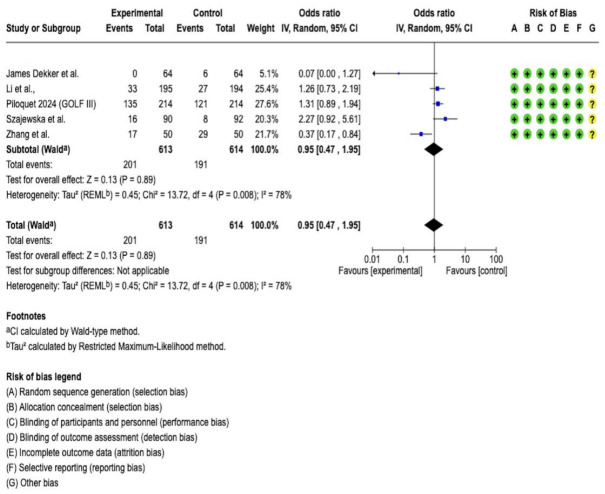
Forest plot of the association between probiotic/synbiotic supplementation and incidence of at least one upper respiratory tract infection (URTI) in children aged ≤24 months. Random-effects meta-analysis of five randomized controlled trials comparing probiotic or synbiotic supplementation with placebo or standard care. Effect estimates are reported as odds ratios (ORs) with 95% confidence intervals (CIs). Squares represent study-specific estimates weighted by inverse variance, and diamonds represent pooled estimates. Heterogeneity was substantial (I^2^ = 78%). The traffic-light symbols displayed on the right side of the forest plot represent study-level risk-of-bias assessments across the evaluated domains [14,28,29,33,34].

**Figure 3:**
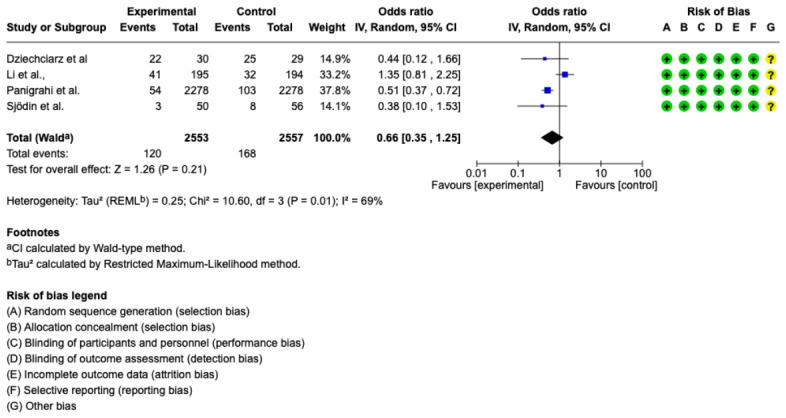
Forest plot of the effect of probiotic/synbiotic supplementation on incidence of any respiratory tract infection (URTI ± LRTI) in children aged ≤24 months. Effect sizes are expressed as odds ratios (ORs) with 95% confidence intervals (CIs) using a random-effects model. Squares represent study-specific estimates weighted by inverse variance, and the diamond represents the pooled effect estimate. Moderate heterogeneity was observed across studies (I^2^ = 69%). The traffic-light symbols displayed on the right side of the forest plot represent study-level risk-of-bias assessments across the evaluated domains [14,28,32,33].

**Figure 4:**
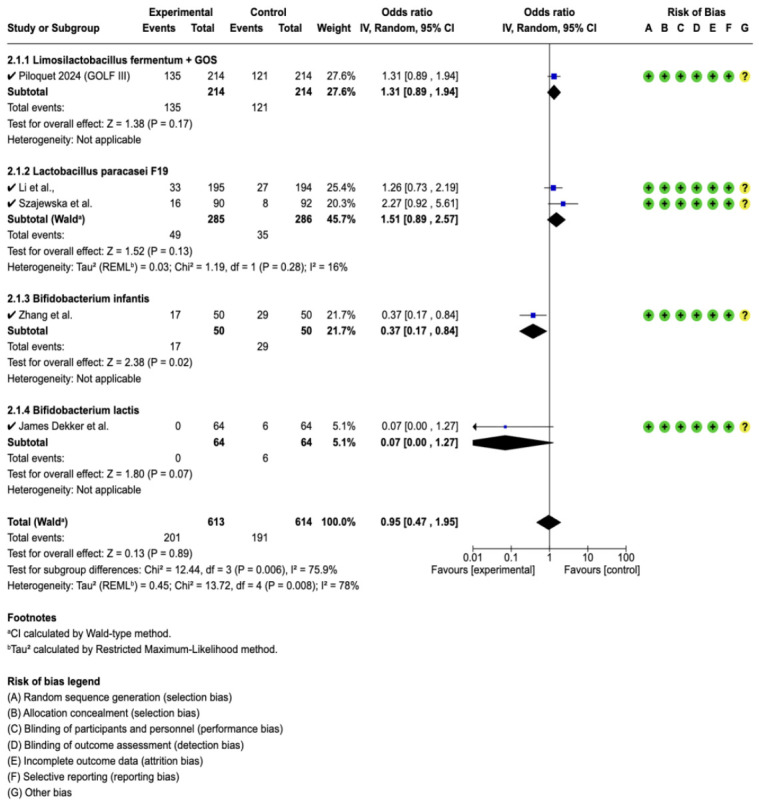
Forest plot of strain-specific subgroup analyses for the incidence of at least one upper respiratory tract infection (URTI) in children aged ≤24 months. Effect estimates are presented as odds ratios (ORs) with 95% confidence intervals (CIs) using a random-effects model. Squares represent study-specific estimates weighted by inverse variance, while diamonds represent pooled subgroup and overall effect estimates. Significant subgroup differences were observed across probiotic strains (χ^2^ = 12.44; *p* = 0.006). The traffic-light symbols displayed on the right side of the forest plot represent study-level risk-of-bias assessments across the evaluated domains [14,28,29,33,34].

**Figure 5:**
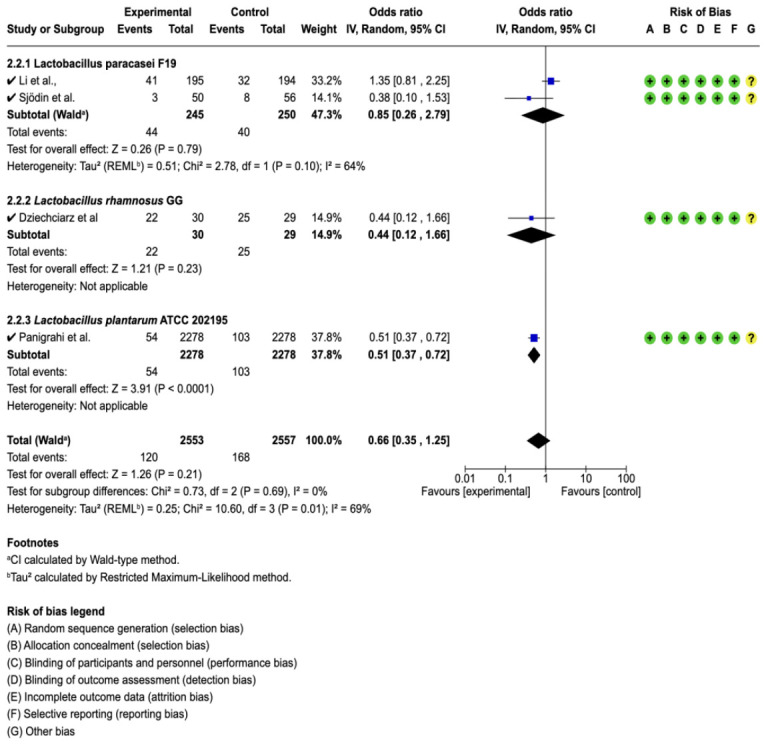
Forest plot of strain-specific subgroup analyses for the incidence of any respiratory tract infection (URTI ± LRTI) in children aged ≤24 months. Effect estimates are presented as odds ratios (ORs) with 95% confidence intervals (CIs) using a random-effects model. Squares represent study-specific estimates weighted by inverse variance, while diamonds represent pooled subgroup and overall effect estimates. No statistically significant subgroup differences were observed across probiotic strains (χ^2^ = 0.73; *p* = 0.69). The traffic-light symbols displayed on the right side of the forest plot represent study-level risk-of-bias assessments across the evaluated domains [5,30,32,33].

According to the Cochrane RoB 2 tool, 5 studies (56%) were judged as having low risk of bias and 4 (44%) as having some concerns, mainly related to allocation concealment or incomplete outcome reporting. No study was rated as hvaing high risk of bias [22]. These ratings were consistent across both primary and secondary outcomes. Study-level risk-of-bias assessments across all evaluated domains are displayed alongside the forest plots (Figure 2, Figure 3, Figure 4 and Figure 5). An overall summary of Cochrane RoB 2 judgments across included studies is provided in Supplementary Figure S1.

### 3.4. Primary Outcome: Incidence of ≥1 Upper Respiratory Tract Infection (URTI)

Five RCTs (n = 1227) reported the incidence of at least one upper respiratory tract infection (URTI) during follow-up. The pooled random-effects analysis showed no significant reduction in risk with probiotic or synbiotic supplementation compared with control: OR = 0.95 [95% CI 0.47–1.95], *p* = 0.89 (I^2^ = 78%, τ^2^ = 0.45) (Figure 2). Certainty of evidence was low due to inconsistency and imprecision.

### 3.5. Secondary Outcome: Incidence of Any Respiratory Infection (URTI ± LRTI)

Four RCTs (n = 5110) reported the incidence of any respiratory tract infection (URTI ± LRTI).

Pooled random-effects analysis showed a non-significant trend toward reduced risk in the probiotic/synbiotic group: OR = 0.66 [95% CI 0.35–1.25], *p* = 0.21 (I^2^ = 69%, τ^2^ = 0.25). (Figure 3). Although the direction of effect was consistent across studies, the wide confidence interval and moderate heterogeneity limited precision. Certainty of evidence was rated moderate (downgraded for imprecision). However, this estimate should be interpreted cautiously, as sensitivity analyses showed that the pooled effect became statistically significant after exclusion of one influential study. No serious adverse events attributable to probiotics were reported across the included studies.

### 3.6. Subgroup Analyses

Upper respiratory tract infections (URTI)

Subgroup analyses of the primary outcome (≥1 URTI) revealed substantial strain-specific variability. *Bifidobacterium longum* subsp. *infantis* demonstrated a statistically significant reduction in URTI incidence (OR = 0.37 [95% CI 0.17–0.84]; *p* = 0.02), indicating a potential protective effect. Strain-specific subgroup analyses for URTI are shown in Figure 4. Conversely, *Limosilactobacillus fermentum* + GOS (OR = 1.31 [0.89–1.94]), *Lactobacillus paracasei* F19 (OR = 1.51 [0.89–2.57]), and *Bifidobacterium animalis* subsp. *lactis* HN019 (OR = 0.07 [0.00–1.27]) did not show statistically significant effects. Between-strain differences were significant (χ^2^ = 12.44; *p* = 0.006), with overall heterogeneity high (I^2^ = 78%). Although the test for subgroup differences was statistically significant, the limited number of studies contributing to each subgroup reduces the robustness of this finding and warrants cautious interpretation. The analysis is underpowered due to the small number of trials contributing to each subgroup. Consequently, these results should be considered exploratory and hypothesis-generating rather than confirmatory evidence of strain-specific efficacy. These findings suggest that probiotic effects on URTI prevention in early life are inconsistent and strongly dependent on strain (Figure 4).

### 3.7. Any Respiratory Infection (URTI ± LRTI)

For the secondary outcome including both upper and lower respiratory tract infections, the pooled random-effects analysis showed no statistically significant difference between probiotic/synbiotic and control groups (OR = 0.66 [95% CI 0.35–1.25]; *p* = 0.21; I^2^ = 69%). No statistically significant subgroup differences were observed across probiotic strains (χ^2^ = 0.73; *p* = 0.69). Subgroup analysis by probiotic strain for any RTI is provided in Figure 5.

*Lactiplantibacillus plantarum* ATCC 202195 demonstrated a significant protective effect (OR = 0.51 [0.37–0.72]; *p* < 0.001), whereas *L. paracasei* F19 (OR = 0.85 [0.26–2.79]) and *L. rhamnosus* GG (OR = 0.44 [0.12–1.66]) showed no significant effects. Overall heterogeneity remained moderate (I^2^ = 69%), and no statistically significant subgroup differences were detected. These results suggest potential strain-specific effects, with *L. plantarum* ATCC 202195 showing the most favorable signal. However, because the estimate was derived from a limited evidence base, the finding should be considered exploratory and requires confirmation in adequately powered randomized trials.

### 3.8. Risk-of-Bias Subgroups

Sensitivity analyses stratified by overall risk of bias (low risk vs. some concerns) showed no meaningful differences in pooled estimates for either URTI or any respiratory infection outcomes.

Effects were consistent across subgroups, and no statistically significant interaction was detected. These findings suggest that methodological quality did not materially influence the observed results, and that residual heterogeneity is more likely attributable to clinical diversity particularly variation in probiotic strain, formulation, population characteristics, and duration of follow-up.

### 3.9. Sensitivity Analyses

Sensitivity analyses were conducted by sequentially excluding individual studies to assess the robustness of pooled estimates. For URTI outcomes, exclusion of Zhang et al. [29] markedly reduced heterogeneity (I^2^ from 84% to 0%) and produced a pooled estimate approaching statistical significance (OR 1.33 [0.99–1.80]; *p* = 0.06), whereas removal of other studies had minimal impact. For any RTI, exclusion of Li et al. [33] reduced heterogeneity (I^2^ from 69% to 0%) and yielded a statistically significant effect (OR 0.50 [0.37–0.69]; *p* < 0.0001); removal of Panigrahi et al. [31] widened CIs and lost significance due to its large weight. Overall, these analyses indicated that some individual studies substantially influenced heterogeneity and, in some cases, the statistical significance of pooled estimates. These findings highlight the sensitivity of the results to specific trials and support cautious interpretation of the pooled effects.

### 3.10. Publication Bias

Publication bias could not be formally assessed because fewer than ten studies contributed to each meta-analysis, and funnel plots or regression-based methods are not reliable under these conditions [26].

### 3.11. Follow-Up Duration

The duration of follow-up across the included RCTs ranged from 2 to 12 months, with a median duration of approximately 3 months (IQR: 3–6 months). Most trials (n = 7) monitored participants for about 3 months, whereas two extended follow-up to 6 or 12 months. This variability in observation period likely contributed to the observed between-study heterogeneity, particularly for dichotomous outcomes such as infection incidence, which are inherently time-dependent. Shorter follow-up trials may have underestimated recurrent infection risk, whereas longer studies captured seasonal fluctuations and provided more complete exposure periods.

### 3.12. Certainty of Evidence (GRADE)

According to the GRADE assessment, the certainty of evidence for the primary outcome (incidence of ≥1 URTI) was rated as low, downgraded for inconsistency (I^2^ = 78%) and imprecision due to wide confidence intervals including the null effect. The certainty of evidence was moderate for the secondary outcome. For the key secondary outcome (any respiratory infection, URTI ± LRTI), the certainty was rated as moderate, downgraded only for imprecision. Although heterogeneity was moderate (I^2^ = 69%), the direction of effect was consistent across all included trials, and variability was mainly attributable to differences in effect magnitude rather than direction; therefore, inconsistency was not downgraded. No serious concerns were identified for risk of bias or indirectness, as most included trials were well-conducted randomized studies in relevant early-life populations. The available evidence indicates that probiotic or synbiotic supplementation during the first two years of life may be associated with a possible reduction in respiratory infections, although the pooled effects were not statistically significant and heterogeneity remains substantial. The observed strain-specific patterns highlight the need for targeted, adequately powered RCTs focusing on individual probiotic formulations in early infancy.

## 4. Discussion

This systematic review and meta-analysis is, to our knowledge, the first to evaluate probiotic and synbiotic supplementation exclusively in infants aged ≤ 24 months. Contrary to previous meta-analyses suggesting a general protective effect of probiotics against respiratory infections in children, our pooled analyses did not demonstrate a statistically significant reduction in the risk of upper respiratory tract infections (URTIs) or any respiratory infection (URTI ± LRTI) in this early-life population. However, exploratory strain-specific signals were observed for *Bifidobacterium longum* subsp. *infantis* and *Lactiplantibacillus plantarum* ATCC 202195. Given the limited number of studies contributing to these analyses and the absence of significant effects in the primary pooled analyses, these findings should be interpreted cautiously and viewed as hypothesis-generating. The certainty of evidence was low for the primary outcome and moderate for the secondary outcome, reflecting inconsistency and imprecision across studies. The observed heterogeneity is biologically plausible given early-life immune and microbial characteristics. Several factors likely contributed to the substantial heterogeneity observed. Specifically, heterogeneity likely arose from differences in participant characteristics (including age at enrollment, feeding modality, and baseline health status), probiotic formulations, administered doses, duration of supplementation, and definitions used to diagnose respiratory infections. These methodological and clinical differences may have influenced both the magnitude and direction of treatment effects across studies. Included studies differed in probiotic strain, dosage, formulation, treatment duration, feeding patterns, baseline microbiota composition, geographical setting, and outcome definitions. The reduction in heterogeneity observed during leave-one-out analyses further suggests that a small number of influential studies contributed disproportionately to between-study variability. From a clinical perspective, this heterogeneity likely reflects meaningful differences in probiotic formulation, administered dose, duration of supplementation, timing of intervention, and baseline population characteristics. Such variability may influence both microbial colonization and host immune responses, potentially explaining the inconsistent treatment effects observed across studies. During early life, the gut microbiota plays a central role in immune development and mucosal regulation [35,36,37]. Disruptions in early colonization (e.g., cesarean delivery, antibiotic exposure, or lack of breastfeeding), have been linked to increased susceptibility to respiratory infections through impaired mucosal immunity [38,39]. Through the gut–lung axis, microbial signals may modulate immune responses [40,41], providing a mechanistic basis for the potential effects of probiotics [42]. The strain-specific patterns observed in this meta-analysis may be consistent with known biological differences among probiotic species. The apparent benefits observed with B. infantis may reflect its unique adaptation to the infant gut through efficient utilization of human milk oligosaccharides and promotion of regulatory immune pathways. Similarly, *L. plantarum* ATCC 202195 has been associated with enhanced innate immune responses and improved mucosal barrier function, mechanisms that may contribute to reduced susceptibility to respiratory infections. Randomized trials have reported protective effects for *Bifidobacterium longum* subsp. *infantis* and *Lactiplantibacillus plantarum* ATCC 202195, whereas other commonly used strains such as *Lactobacillus paracasei* F19 and *Lactobacillus rhamnosus* GG have not demonstrated consistent benefit [29,31,33]. Infant-type bifidobacteria, particularly *B. infantis*, are well adapted to the breastfed infant gut and may enhance barrier function and immune regulation, potentially reducing respiratory susceptibility via the gut–lung axis [43,44]. In contrast, the colonization dynamics of other Lactobacillus species may be less stable in early infancy, which could partly explain the weaker or inconsistent effects observed [45]. An important limitation of the present review is the limited statistical power of the strain-specific subgroup analyses. Although restricting the analysis to children aged ≤24 months allowed us to address an important gap in the literature and focus on a biologically distinct developmental period, this approach inevitably reduced the number of eligible studies. As a result, several strain-specific subgroup analyses were based on a small number of trials, limiting statistical power and increasing uncertainty around subgroup estimates. Most subgroups were informed by only one or two studies, making the corresponding estimates vulnerable to random error and reducing confidence in between-strain comparisons. Therefore, although certain strains demonstrated potentially favorable effects, these observations should be regarded as exploratory and hypothesis-generating rather than definitive evidence of superiority. This may explain why trials in older children show more consistent effects. Current evidence does not support routine probiotic administration for respiratory infection prevention in infants. Safety considerations are also relevant when evaluating probiotic supplementation in early life. Although no serious adverse events attributable to probiotics or synbiotics were reported in the included trials, safety cannot be assumed across all infant populations. Rare cases of probiotic-associated sepsis have been described, particularly among preterm infants, very-low-birth-weight neonates, and severely immunocompromised patients. Therefore, while the available evidence suggests an overall favorable safety profile in healthy infants, caution remains warranted in vulnerable populations, and future trials should continue to systematically assess and report safety outcomes. However, the observed strain-specific signals suggest that targeted supplementation strategies may be beneficial in selected populations, particularly in contexts characterized by altered microbiome development, such as antibiotic exposure, cesarean delivery, or formula feeding [35,36]. Future randomized trials should therefore adopt strain-specific and context-aware designs, with rigorous characterization of probiotic interventions and adequate follow-up to capture seasonal and developmental variability. This study has several strengths. It is the first meta-analysis restricted to infants ≤24 months and includes only prophylactic randomized controlled trials. The protocol was pre-registered (PROSPERO: CRD4202021157621) and conducted according to PRISMA 2020 guidelines [20]. Methodological rigor was further supported by the use of the Cochrane RoB 2 tool and the GRADE framework [22,27]. In addition, strain-level subgroup analyses allowed identification of potential signal effects that would likely be obscured in pooled genus-level estimates. Several limitations should be acknowledged. Significant between-study heterogeneity was observed, likely reflecting differences in probiotic strains, formulations, doses, timing of administration, population characteristics, feeding practices, and outcome definitions. The delivery vehicles used across studies also varied and included infant formula, powders, capsules, and sachets. Although a formal subgroup analysis according to delivery vehicle was not feasible, no obvious pattern suggesting differential efficacy according to the method of administration was observed. Outcome ascertainment also varied across studies. While some trials relied on physician-confirmed diagnoses of respiratory infections, others incorporated parent-reported symptoms or caregiver-completed illness records. This variability may have introduced outcome misclassification and contributed to the observed heterogeneity across studies. Although feeding modality is a major determinant of infant microbiota composition and immune development, stratified analyses according to breastfeeding status were not feasible because these data were inconsistently reported across the included studies. Consequently, it was not possible to determine whether probiotic efficacy differed between exclusively breastfed infants and those receiving formula feeding. Geographical variability may also have contributed to the observed heterogeneity. The included trials were conducted across Europe and Asia, where baseline microbiota composition, dietary habits, environmental exposures, healthcare practices, and circulating respiratory pathogens may differ substantially. These factors could influence both susceptibility to respiratory infections and responsiveness to probiotic supplementation, potentially contributing to the variability of treatment effects observed across studies. Dose–response analyses were not feasible because probiotic doses varied substantially across studies and were frequently reported using non-comparable formulations, preventing reliable quantitative assessment of dosage effects. The limited number of trials available for each probiotic strain reduced the precision of subgroup estimates and precluded robust strain-specific meta-analyses. Consequently, the observed strain-specific effects should be interpreted with caution because they were frequently based on single studies or very small numbers of participants. Reporting of secondary outcomes, including infection severity, duration, healthcare utilization, absenteeism, and antibiotic use, was inconsistent across studies. Definitions, measurement methods, and reporting formats varied substantially, preventing quantitative synthesis and limiting the ability to determine whether probiotic supplementation influenced clinically relevant outcomes beyond infection incidence. Restriction to English-language publications may have introduced language bias, and sensitivity analyses indicated that individual studies influenced heterogeneity and pooled estimates. These limitations highlight the need for larger, standardized trials. Future studies should adopt harmonized outcome definitions, standardized reporting of secondary clinical endpoints, and adequately powered strain-specific designs to improve the reliability and interpretability of evidence in this field. Consequently, the observed strain-specific findings should be considered exploratory and hypothesis-generating rather than definitive evidence of efficacy.

### 4.1. Clinical Implication

Although current evidence does not support routine probiotic supplementation for respiratory infection prevention in healthy infants, certain subgroups may deserve particular attention. From a health-economic perspective, routine probiotic administration cannot currently be considered cost-effective given the absence of a consistent preventive effect. Future economic evaluations should focus on high-risk infant populations and on strains demonstrating the strongest efficacy signals. Infants born by cesarean section, exposed to antibiotics during early life, or predominantly formula-fed frequently exhibit altered microbiota development and may represent populations at increased risk of respiratory infections. In the present review, *Bifidobacterium longum* subsp. *infantis* and *Lactiplantibacillus plantarum* ATCC 202195 showed the most promising strain-specific signals. However, given the low-to-moderate certainty of evidence and the limited number of available studies, these findings should be considered hypothesis-generating rather than sufficient to support routine clinical recommendations.

### 4.2. Future Directions

These findings support a precision-based research approach, as no overall preventive effect was observed despite potential strain-specific benefits. Future randomized controlled trials should adopt harmonized methodological standards to improve comparability across studies. Standardized definitions of upper respiratory tract infections (URTI) and respiratory tract infections (RTI), together with clinically confirmed outcome measures, should be used whenever possible. Probiotic interventions should be reported in detail, including strain identification, formulation, dose (CFU/day), timing of administration, and duration of supplementation. Follow-up periods should ideally extend across at least one complete respiratory infection season to adequately capture infection risk and seasonal variability. Future studies should also incorporate predefined population stratification according to factors known to influence early microbiota development, including mode of delivery, antibiotic exposure, and feeding practices. Particular attention should be given to high-risk infant populations, such as those born by cesarean section, exposed to antibiotics during early life, or predominantly formula-fed. In addition to infection incidence, future trials should standardize the reporting of clinically relevant secondary outcomes, including infection frequency, duration, severity, healthcare utilization, absenteeism, and antibiotic consumption. These methodological improvements may help identify which probiotic strains, doses, and intervention strategies are most effective for respiratory infection prevention during the first two years of life.

## 5. Conclusions

This review found no convincing evidence that probiotic or synbiotic supplementation reduces respiratory infections in infants when all strains are considered together. Our findings indicate that early-life probiotic prophylaxis does not confer a consistent or statistically significant reduction in respiratory infection risk at the population level. However, exploratory subgroup analyses identified potentially favorable signals for *Bifidobacterium longum* subsp. *infantis* and *Lactiplantibacillus plantarum* ATCC 202195. Given the limited number of contributing studies and the lack of statistically significant effects in the primary pooled analyses, these findings should be considered hypothesis-generating and require confirmation in adequately powered randomized controlled trials. These findings may be particularly relevant for infants at increased risk of microbiota disruption, including those born by cesarean section, exposed to antibiotics, or predominantly formula-fed, although current evidence remains insufficient to support routine clinical implementation. These results contribute to the growing body of evidence supporting the investigation of precision-based, strain-specific approaches rather than generalized probiotic supplementation. Further large, well-designed randomized trials are needed to define which probiotic strategies may be effective for respiratory infection prevention in early life.

## Figures and Tables

**Table 1:** Characteristics of the randomized controlled trials included in the systematic review and meta-analysis.

Study	Country	Age Range	N (Intervention)	N (Control)	Probiotic Strain	Comparator	Follow-Up (Months)	Outcome
Piloquet 2024 (GOLF III) [28]	France, Belgium	4 weeks ± 1 week	214	214	*Limosilactobacillus fermentum* CECT5716 + GOS (synbiotic)	Standard infant & follow-on formula without synbiotics (placebo)	12	URTI
Zhang et al. [29]	China	0–36 months	50	50	*Bifidobacterium longum* subsp. *infantis* YLGB-1496	Placebo (maltodextrin)	3	URTI
Szajewska et al. [14]	Poland	≤28 days (term infants, 38–42 weeks gestation, birth weight 2700–4200 g)	90	92	*Lactobacillus paracasei* ssp. *paracasei* F19	Prebiotic formula (FOS/GOS only)	12	URTI
Sjödin et al. [30]	Poland	≤28 days at enrolment; term infants 38–42 weeks GA, BW 2700–4200 g.	50	56	*Lactobacillus paracasei* ssp. *paracasei* F19	Prebiotic formula (FOS/GOS only)	12	Any RTI
Panigrahi et al. [31]	India	0–4 days (≥35 weeks gestation, ≥2000 g birth weight)	2278	2278	*Lactiplantibacillus plantarum* ATCC 202195	Placebo (maltodextrin)	2	Any RTI
Dziechciarz et al. 2020 (LGG in children on PPI) [32]	Poland (Medical University of Warsaw)	0.5–48 months overall (placebo 0.5–48; LGG 2–15)	30	29	*Lactobacillus rhamnosus* GG	Placebo (identical in appearance and taste)	12	Any RTI
Li et al. [33]	China	≤1 month at enrollment	195	194	*Lactobacillus paracasei* ssp. *paracasei* F19	Standard infant formula without probiotic	4	URTI
Li et al. [33]	China	≤1 month at enrollment	195	194	*Lactobacillus paracasei* ssp. *paracasei* F19	Standard infant formula without probiotic	4	Any RTI
James Dekker et al. [34]	China	6–12 months	64	64	*Bifidobacterium animalis* subsp. *lactis* HN019	placebo	3	URTI

Abbreviations: URTI, upper respiratory tract infection; RTI, respiratory tract infection; GOS, galacto-oligosaccharides; FOS, fructo-oligosaccharides; GA, gestational age; BW, birth weight; PPI, proton pump inhibitor; LGG, *Lactobacillus rhamnosus* GG; N, number of participants.

## Data Availability

No new data were created or analyzed in this study. Data sharing is not applicable to this article.

## Supplementary Materials

The following supporting information can be downloaded at: https://www.mdpi.com/article/10.3390/nu18132067/s1. Figure S1: Risk-of-bias assessment of included randomized controlled trials using the Cochrane RoB 2 tool.

## Abbreviations

The following abbreviations are used in this manuscript:

RTI	Respiratory tract infection
URTI	Upper respiratory tract infection
LRTI	Lower respiratory tract infection
RCT	Randomized controlled trial
PRISMA	Preferred Reporting Items for Systematic Reviews and Meta-Analyses
GRADE	Grading of Recommendations Assessment, Development and Evaluation
RoB	Risk of bias
CI	Confidence interval
OR	Odds ratio
GOS	Galacto-oligosaccharides
FOS	Fructo-oligosaccharides
PPI	Proton pump inhibitor
