# Supplementary material for: Strain-Specific Effects of Early-Life Probiotic Supplementation on Respiratory Infections in Infants: A Systematic Review and Meta-Analysis
Source: Nutrients. 2026 Jun 24;18(13):2067. doi: 10.3390/nu18132067 (PMC13363551; doi:10.3390/nu18132067)
Supplement: Supplementary file 1 [file nutrients-18-02067-s001.zip › nutrients-4365980-supplementary.pdf]

Supplementary Figure S1

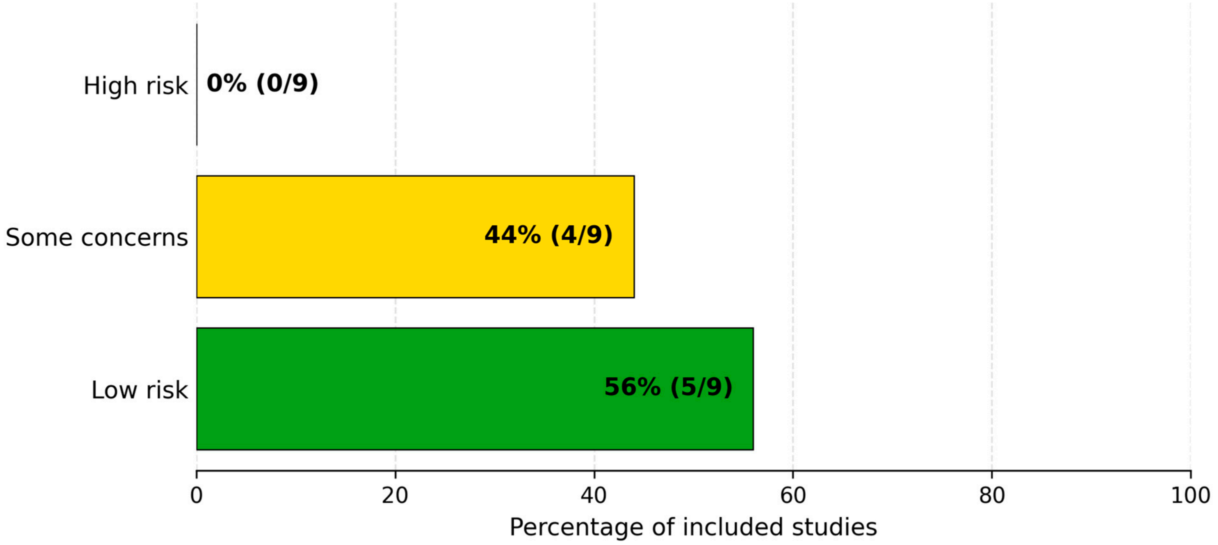

Figure S1. Overall distribution of Cochrane RoB 2 judgments across included randomized controlled trials (n = 9). Five studies (56%) were rated as low risk of bias, four studies (44%) were rated as having some concerns, and no study was judged at high risk of bias.
